# Imputation of *TPMT* defective alleles for the identification of patients with high-risk phenotypes

**DOI:** 10.3389/fgene.2014.00096

**Published:** 2014-05-12

**Authors:** Berta Almoguera, Lyam Vazquez, John J. Connolly, Jonathan Bradfield, Patrick Sleiman, Brendan Keating, Hakon Hakonarson

**Affiliations:** ^1^Center for Applied Genomics, The Children's Hospital of PhiladelphiaPhiladelphia, PA, USA; ^2^Department of Pediatrics, University of Pennsylvania Perelman School of MedicinePhiladelphia, PA, USA

**Keywords:** TPMT, genotype imputation, DNA biobank, pharmacogenetics, Electronic Medical Records

## Abstract

**Background**: The activity of thiopurine methyltransferase (TPMT) is subject to genetic variation. Loss-of-function alleles are associated with various degrees of myelosuppression after treatment with thiopurine drugs, thus genotype-based dosing recommendations currently exist. The aim of this study was to evaluate the potential utility of leveraging genomic data from large biorepositories in the identification of individuals with TPMT defective alleles.

**Material and methods**: *TPMT* variants were imputed using the 1000 Genomes Project reference panel in 87,979 samples from the biobank at The Children's Hospital of Philadelphia. Population ancestry was determined by principal component analysis using HapMap3 samples as reference. Frequencies of the TPMT imputed alleles, genotypes and the associated phenotype were determined across the different populations. A sample of 630 subjects with genotype data from Sanger sequencing (*N* = 59) and direct genotyping (*N* = 583) (12 samples overlapping in the two groups) was used to check the concordance between the imputed and observed genotypes, as well as the sensitivity, specificity and positive and negative predictive values of the imputation.

**Results**: Two SNPs (rs1800460 and rs1142345) that represent three *TPMT* alleles (^*^3A, ^*^3B, and ^*^3C) were imputed with adequate quality. Frequency for the associated enzyme activity varied across populations and 89.36–94.58% were predicted to have normal TPMT activity, 5.3–10.31% intermediate and 0.12–0.34% poor activities. Overall, 98.88% of individuals (623/630) were correctly imputed into carrying no risk alleles (553/553), heterozygous (45/46) and homozygous (25/31). Sensitivity, specificity and predictive values of imputation were over 90% in all cases except for the sensitivity of imputing homozygous subjects that was 80.64%.

**Conclusion**: Imputation of TPMT alleles from existing genomic data can be used as a first step in the screening of individuals at risk of developing serious adverse events secondary to thiopurine drugs.

## Introduction

Thiopurine S-methyltransferase (TPMT) is an enzyme involved in the metabolism of purine analogs such as azathioprine, 6-mercaptopurine and thioguanine, drugs that are used as chemotherapeutic and immunosuppressant agents in diseases such as lymphoid malignancies, leukemias, inflammatory bowel disease, and other immune conditions (Relling et al., 2011; Appell et al., 2013). *TPMT* maps to chromosome 6p22.3. It is subject to genetic variation and, to date, 34 alleles have been identified and characterized, most of which are associated with reduced activity *in vitro* (Relling et al., 2011). Alleles ^*^2 (rs1800462), ^*^3A (rs1800460 and rs1142345), ^*^3B (rs1800460), and ^*^3C (rs1142345) account for 95% of all defective alleles and all four involve missense mutations: allele ^*^2 results in the p.Ala80Pro change (chr6:18143955), allele ^*^3A contains two missense changes: p.Ala154Thr (chr6: 6:18139228) and p.Tyr240Cys (chr6:18130918), and alleles ^*^3B and ^*^3C are defined by p.Ala154Thr and p.Tyr240Cys, respectively (reference sequence NP_000358.1). The frequencies of these alleles vary significantly across ethnic populations (Appell et al., 2013): while ^*^3A is the most frequently found in Caucasians (4.5%) (Schaeffeler et al., 2004), ^*^3C is more prevalent in Africans or Asians, with 5.4–7.6% and 0.3–3%, respectively (reviewed in Teml et al., 2007).

TPMT enzymatic activity exhibits a trimodal distribution and approximately 0.3% of the population carry two defective alleles (associated with negligible activity), about 10% are heterozygous (intermediate activity), and 89% have normal activity (Weinshilboum and Sladek, 1980; Schaeffeler et al., 2004). Therefore, both heterozygous and homozygous individuals are at higher risk of developing myelosuppression within a few weeks after starting treatment with conventional doses that can be lethal if unrecognized, independent of the underlying disease being treated (Sim et al., 2013).

Due to the potential cytotoxicity and narrow therapeutic index of thiopurines, the US Food and Drug Administration (FDA) recommends *TPMT* testing prior to starting treatment with thiopurine drugs, and *TPMT* genotype-guided dosing recommendations are currently in use (Relling et al., 2011, 2013).

Results of genetic tests are potentially relevant over a patient's lifetime and having that information incorporated into patients' medical records may be useful in the improvement and guidance of drug treatments, if ever needed. With electronic medical records (EMR) currently widely implemented at academic hospitals and other treatment institutions, pharmacogenetic actionable variants can be integrated to the already available patient's information, helping optimize clinical decision making and care planning (Gottesman et al., 2013). Moreover, genome-wide data is increasingly accessible due to decreasing costs of genomic technologies and the development of methods that allow for accurate imputation of genotypes not directly probed by specific arrays could influence health care decisions (Marchini and Howie, 2010). Genomic data is frequently stored within large biorepositories where DNA samples are linked with phenotypic data. These biorepositories have been efficient and successful in studies of genotype-phenotype associations and they can be used as a model for the implementation and evaluation of pharmacogenomics in routine clinical practice (Gottesman et al., 2013).

In the present study, we leverage existing genome-wide genotyping data to impute common defective *TPMT* alleles with the aim of identifying individuals carrying high-risk genotypes for thiopurines-induced adverse events.

## Material and methods

### Subjects and genotyping

This study was approved by the institutional review board and the ethics committee of The Children's Hospital of Philadelphia (CHOP). Written informed consent was obtained from each participant in accordance with institutional requirements and the Declaration of Helsinki Principles. Subjects were selected from the biorepository at the Center for Applied Genomics at CHOP. The CHOP biobank has a collection of over 160,000 samples including 60,000 internal pediatric samples and over 100,000 adult and pediatric samples from external collaborators genotyped using standard GWAS arrays from Illumina and Affymetrix (summarized in Gottesman et al., 2013).

**Figure 1:**
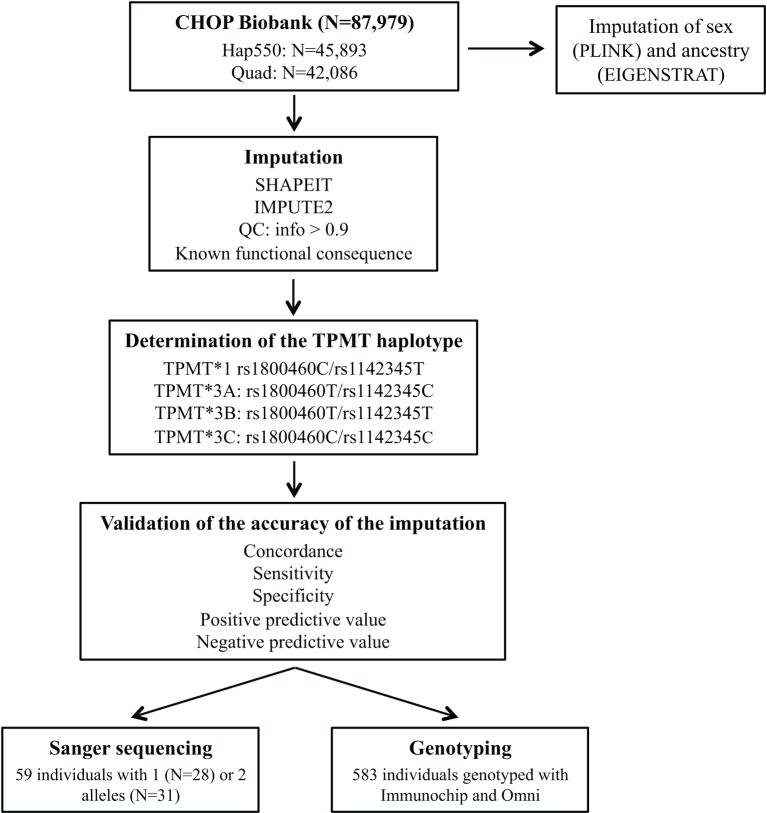
**General schema for the study process**. Hap550 is the InfiniumII HumanHap550 array, Quad is Human610-Quad version 1 array, Immunochip is the Illumina Infinium Immunochip array and Omni is the HumanOmni1-Quad version 1.

Figure 1 illustrates the study process. For *TPMT* imputation, we selected a total of 87,979 samples genotyped with either InfiniumII HumanHap550 (550; *N* = 45,893) or Human610-Quad version 1 (Quad; *N* = 42,086) arrays (Illumina, San Diego, CA). Genotyping data were used to impute sex using PLINK (http://pngu.mgh.harvard.edu/purcell/plink/) (Purcell et al., 2007); population ancestry was determined by principal component analysis (Eigenstrat 3.0) (Price et al., 2006), and samples were grouped into populations using nearest neighbors analysis and the HapMap3 samples (https://www.sanger.ac.uk/resources/downloads/human/hapmap3.html) as a reference.

### Imputation of TPMT genotypes

Imputation of unobserved genotypes in *TPMT* gene locus (chr6:18,128,545–18,155,374) was carried out with the IMPUTE2 package (http://mathgen.stats.ox.ac.uk/impute/impute_v2.html) (Howie et al., 2009) with the 1,000 Genomes Project reference panel, after prephasing chromosome 6 haplotypes with SHAPEIT version 2 (http://www.shapeit.fr/) (Delaneau et al., 2013). Since rs1800460 is probed on the Illumina HH610 Quad array, prephasing and imputation was performed for each chip type separately. Quality control filters were applied and only SNPs with an info score >0.9 were kept.

### Validation of the imputed genotypes

To determine the accuracy of imputation, *TPMT* imputed haplotypes were compared to those obtained by other genotyping platforms covering *TPMT* variation. Of the 87,979 samples, 583 also had genotyping data on Illumina Infinium Immunochip (Immunochip), and HumanOmni1-Quad version 1 (Omni), which captured both rs1800460 and rs1142345. Additionally, Sanger sequencing of rs1800460 in exon 7 and rs1142345 in exon 10 was used to validate the imputation results (primers previously described in Schaeffeler et al., 2001). The sample selected for Sanger sequencing consisted of 59 individuals predicted to carry one or two defective alleles by imputation that had been exposed to a TPMT medication based on the EMR.

### Determination of the concordance and the sensitivity, specificity, positive and negative predictive values of the imputation for the identification of carriers of TPMT defective alleles

**Figure 2:**
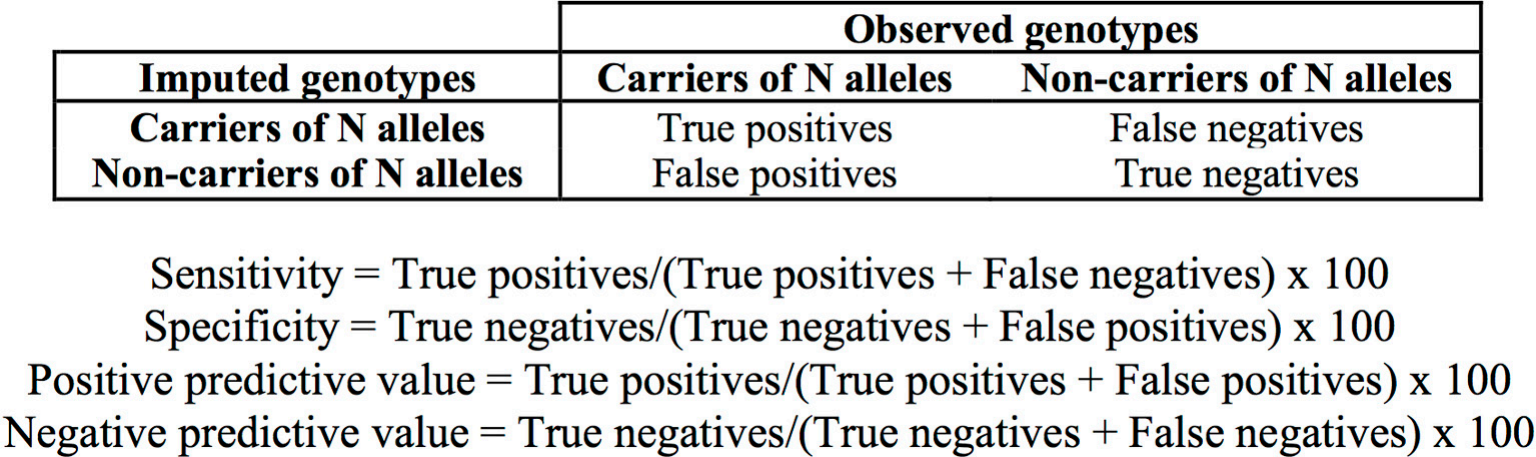
**Definition of true and false positive and negative values and formulae used for the determination of the sensitivity, specificity, and predictive values of the imputation**.

We determined the concordance of the imputation as the number of imputed genotypes that correspond with output from direct genotyping or sequencing (expressed as percentage). Sensitivity, specificity and positive and negative predictive values of the imputation in the discrimination of subjects carrying the ^*^1/^*^1 genotype, and one and two *TPMT* defective alleles were determined as shown in Figure 2.

## Results

### Subjects and TPMT imputation

Ancestry, sex estimation and imputation of *TPMT* genotypes for the 89,797 individuals were performed in eleven batches of ~8,000 samples. An average of 174,911 SNPs with *r*^2^ < 0.2 were used for principal components calculation.

There were 50.04% males out of the 89,797 individuals (imputed sex for 0.87% of the individuals was undetermined). Principal component analysis classified 72.74% of individuals as Caucasians, 18.78% with African ancestry, 6.55% Hispanics, and 1.93% Asians (Table 1). There were also data on self-reported ethnicity for 24,527 out of the 89,797 individuals, with 50.15% Caucasians (*N* = 12,304), 41.81% African Americans (*N* = 10,263), 1.6% Asians (*N* = 385), 0.08% American Indians (*N* = 21), 0.03% Native Hawaiians (*N* = 7), 0.02% Indians (*N* = 5), and 6.29% were considered as “Other” (*N* = 1542). Concordance between self-reported and imputed ancestry was >80% for Caucasians, African Americans, and Asians (93.92, 98.34, and 81.30%, respectively). For the remaining groups, 57.58% were classified as Hispanics (19 out of 33), 15.15% as Asians (*N* = 5) and Caucasians (*N* = 5) and 12.12% as African Americans (*N* = 4).

**Table 1:** **Frequencies of the different ethnicities in the sample investigated based on principal component analysis of imputed genotype data**.

**Population**	***N***	**(%)**
**Caucasians**	63,998	72.74
CEU, Utah residents with ancestry from northern and western Europe	56,675	64.42
TSI, Toscans in Italy	7,323	8.32
African ancestry	16,519	18.78
ASW, African ancestry in Southwest USA	15,457	17.57
YRI, Yoruba in Ibadan, Nigeria	947	1.08
MKK, Maasai in Kinyawa, Kenya	96	0.11
LWK, Luhya in Webuye, Kenya	19	0.02
**Hispanics**	5,764	6.55
MEX, Mexican ancestry in Los Angeles, California	4,786	5.44
GIH, Gujarati Indians in Houston, Texas	978	1.10
**Asians**	1,698	1.93
CHD, Chinese in Metropolitan Denver, Colorado	1,043	1.19
JPT, Japanese in Tokyo, Japan	269	0.91
CHB, Han Chinese in Beijing, China	386	0.43

Three hundred and fifty four variants were imputed in the *TPMT* gene, including 322 SNPs and 33 insertion/deletion polymorphisms (*indels*). Out of these, only 117 had an info value ≥0.9 (103 SNPs and 14 *indels*). However for the subsequent analysis only those with a known functional significance were considered: rs1800460 and rs1142345, which define alleles ^*^3A, ^*^3B, and ^*^3C. The loss-of-function variant rs1800462 that defines allele ^*^2 was also imputed but did not pass the quality filters, thus it was excluded from the further analyses.

*TPMT* alleles were assigned as ^*^1 when the rs1800460 C>T/rs1142345 T>C diplotype was CT, ^*^3B when TT, and ^*^3C when CC. For allele ^*^3A, given the high linkage disequilibrium between rs1800460 (^*^3B) and rs1142345 (^*^3C) and the low minor allele frequency, whenever an individual carried both variants (rs1800460T and rs1142345C), the allele was assigned as ^*^3A.

*TPMT* allelic, genotypic and associated phenotypic frequencies for ^*^3A, ^*^3B, and ^*^3C across ethnic groups are illustrated in Tables 2, 3 and 4, respectively. As shown in Table 2, the distribution of the three defective alleles varied largely across populations: ^*^3A was more represented among the Caucasians, ^*^3B in the Hispanics and ^*^3C in both Asians and African Americans, being the latter the group with the highest frequency of carriers of *TPMT* defective alleles (5.49 vs. 4.07% in Caucasians, 4.41% in Hispanics and 2.77% in Asians). According to the genotype-associated enzymatic activity, Asians harbored the lowest rates of poor metabolizers with only 0.12% whereas Caucasians, African Americans and Hispanics have a frequency close to 0.33%.

**Table 2:** Distribution of allele frequencies for *TPMT* alleles ^*^3A, ^*^3B, and ^*^3C across the different ethnic groups.

	**Caucasian (*N* = 63,998)**	**AA (*N* = 16,519)**	**Hispanic (*N* = 5,764)**	**Asian (*N* = 1,698)**	**Total (*N* = 87,979)**
**Allele**	***N* (%)**	***N* (%)**	***N* (%)**	***N***	***N* (%)**
^*^1	122,787 (95.93)	31,225 (94.51)	11,020 (95.59)	3,302 (97.23)	168,333 (95.67)
^*^3A	4,305 (3.36)	303 (0.92)	334 (2.90)	19 (0.56)	4,961 (2.82)
^*^3B	86 (0.07)	1 (0.00)	12 (0.10)	0 (0.00)	99 (0.06)
^*^3C	817 (0.64)	1,509 (4.57)	162 (1.41)	75 (2.21)	2,563 (1.46)

**Table 3:** Distribution of genotypes for *TPMT* alleles ^*^3A, ^*^3B, and ^*^3C across the different ethnic groups.

	**Caucasian (*N* = 63,998)**	**AA (*N* = 16,519)**	**Hispanic (*N* = 5,764)**	**Asian (*N* = 1,698)**	**Total (*N* = 87,979)**
**Genotype**	***N* (%)**	***N* (%)**	***N* (%)**	***N* (%)**	***N* (%)**
^*^1/^*^1	58,981 (92.16)	14,761 (89.36)	5,275 (91.52)	1,606 (94.58)	80,623 (91.64)
^*^1/^*^3A	4,119 (6.44)	286 (1.73)	322 (5.59)	19 (1.12)	4,746 (5.39)
^*^1/^*^3B	10 (0.02)	1 (0.01)	1 (0.02)	0 (0.00)	12 (0.01)
^*^1/^*^3C	697 (1.09)	1,416 (8.57)	147 (2.55)	71 (4.18)	2,331 (2.65)
^*^3A/^*^3A	81 (0.13)	1 (0.01)	5 (0.09)	0 (0.00)	87 (0.10)
^*^3A/^*^3B	1 (0.01)	0 (0.00)	0 (0.00)	0 (0.00)	1 (0.001)
^*^3A/^*^3C	23 (0.04)	15 (0.09)	2 (0.03)	0 (0.00)	40 (0.05)
^*^3B/^*^3C	75 (0.12)	0 (0.00)	11 (0.19)	0 (0.00)	86 (0.10)
^*^3C/^*^3C	11(0.02)	39 (0.24)	1 (0.02)	2 (0.12)	53 (0.06)

**Table 4:** ***TPMT* genotype-associated phenotypic frequencies across the different ethnic groups**.

	**Caucasian (*N* = 63,998)**	**AA (*N* = 16,519)**	**Hispanic (*N* = 5,764)**	**Asian (*N* = 1,698)**	**Total (*N* = 87,979)**
**TPMT activity**	***N* (%)**	***N* (%)**	***N* (%)**	***N* (%)**	***N* (%)**
Normal	58,981 (92.16)	14,761 (89.36)	5,275 (91.52)	1,606 (94.58)	80,623 (91.64)
Intermediate	4,826 (7.54)	1,703 (10.31)	470 (8.15)	90 (5.30)	7,089 (8.06)
Low	191 (0.30)	55 (0.33)	19 (0.33)	2 (0.12)	267 (0.30)

### Concordance, sensitivity, specificity, and positive and negative predictive values of the imputation

Out of the 87,979 samples used for imputation, 583 had genotyping data on both rs1800460 and rs1142345: 94.8% of them carried the genotype ^*^1/^*^1, 4.5% ^*^1/^*^3A, and 0.7% the genotype ^*^1/^*^3C. Concordance of the imputed haplotypes compared to those determined by genotyping was 99.8% (Table 5).

**Table 5:** **Concordance between the imputed genotypes and genotypes determined by genotyping using Immunochip (Illumina Infinium Immunochip array) and Omni (HumanOmni1-Quad version 1) (*N* = 583)**.

**Imputation**	**Genotyping**			
	^*^1/^*^1	^*^1/^*^3A	^*^1/^*^3C	**Total**
^*^1/^*^1	553	0	0	553
^*^1/^*^3A	0	26	0	26
^*^1/^*^3C	1	0	3	4
Total	554	26	3	583

Sanger sequencing was performed in a subset of 59 samples predicted to carry 1 (*N* = 28) or 2 (*N* = 31) defective alleles (^*^3A, or ^*^3C). Twelve of the 59 samples also had genotype data and results were consistent across the two methods and with the imputation. The overall concordance was 84.7% for the total 59 samples. Table 6 illustrates the concordance of the imputed genotypes after validation with Sanger sequencing.

**Table 6:** **Concordance between imputed genotypes and genotypes determined by Sanger sequencing (*N* = 59)**.

**Imputation**	**Sanger sequencing**						
	^*^1/^*^1	^*^1/^*^3A	^*^1/^*^3C	^*^3A/^*^3A	^*^3A/^*^3C	^*^3C/^*^3C	**Total**
^*^1/^*^1	0	0	0	0	0	0	0
^*^1/^*^3A	0	19	0	0	0	0	19
^*^1/^*^3C	0	0	9	0	0	0	9
^*^3A/^*^3A	1	0	0	6	0	0	7
^*^3A/^*^3C	0	0	1	0	3	1	5
^*^3C/^*^3C	0	0	4	1	1	13	19
Total	1	19	14	7	4	14	59

When taking into account the number of defective alleles, 98.88% of individuals (623 of the 630 individuals—excluding the 12 in the two groups-) were accurately imputed. All of the samples identified as carrying no risk alleles (^*^1/^*^1) were confirmed by direct genotyping or sequencing (553/553) and for heterozygous and homozygous individuals, the concordance was lower, with 97.8% (45/46) and 80.64% (25/31), respectively (Table 7). Sensitivity, specificity and positive and negative predictive values of imputation of *TPMT* genotypes are summarized in Table 7. Since the importance of *TPMT* genotyping lies in the discrimination of individuals carrying defective alleles, these metrics were determined for the discrimination of individuals carrying the ^*^1/^*^1 genotype, individuals with one defective allele and individuals with two defective alleles. Sensitivity, specificity, and predictive values were close to 100% for all cases except for the positive predictive value of identifying homozygous carriers, which was 80.64%.

**Table 7:** **Concordance between imputed and observed genotypes according to the number of defective alleles and characteristics of the imputation in terms of sensitivity (S), specificity (SP), positive predictive value (PPV) and negative predictive value (NPV) (*N* = 630)**.

	**Observed genotypes**			**Imputation metrics**			
**Imputed genotypes**	**0 alleles**	**1 allele**	**2 alleles**	**S**	**SP**	**PPV**	**NPV**
0 alleles	553	0	0	99.64	100	100	97.40
1 allele	1	45	0	90.00	99.83	97.82	99.14
2 alleles	1	5	25	100	99.01	80.64	100
Total	555	50	25				

## Discussion

A growing number of drug-gene interactions, affecting routinely prescribed drugs, are being validated (Relling and Klein, 2011). The FDA has to date recommended the inclusion of pharmacogenetic markers in the labels of more than a 100 drugs (http://www.fda.gov/drugs/scienceresearch/researchareas/pharmacogenetics/ucm083378.htm) (Shuldiner et al., 2013) and initiatives such as Pharmacogenomics Knowledge Base (PharmGKB) and the Clinical Pharmacogenetics Implementation Consortium (CPIC) (Relling and Klein, 2011) provide essential pharmacogenetic information and play a major role in establishing recommendations to aid clinicians in guiding therapies. If one considers the report by Schildcrout et al, who demonstrated that up to 65% of patients were exposed to at least one medication with an established drug-gene association within 5 years (Schildcrout et al., 2012), then pharmacogenetics integration into individuals' medical records for clinical use is becoming an urgent need. Availability of pharmacogenetic information prior to patients' treatment has the opportunity to identify individuals potentially benefiting from a given therapy, select adequate medications and doses, in order to ultimately administer the most effective treatment to each patient, with the lower incidence of adverse events.

Using existing genomic data from the CHOP biobank repository we have been able to impute three of the most common defective *TPMT* alleles ^*^3A, ^*^3B, ^*^3C in a cohort of 87,979 individuals. The sensitivity, specificity and positive and negative predictive values of the imputation were sufficiently high to allow discrimination of patients carrying one or two defective alleles from those with a ^*^1/^*^1 genotype. Concordance between observed and imputed genotypes was 100% for individuals with ^*^1/^*^1 genotype, and for carriers of *TPMT* alleles, discordant results were essentially cases of individuals predicted to be heterozygous by imputation that were found to be homozygous by genotyping or sequencing. Additionally, probably because of the rarity of SNPs rs1800460 and rs1142345 and the increase in imputation errors as minor allele frequency decreases, alleles ^*^3A and ^*^3C were frequently switched, and allele ^*^3B was only identified in the subset of samples genotyped with the Quad array. The rarity of allele ^*^2 may also be the explanation for the inability of imputing with adequate quality.

TPMT deficiency exhibits an extensive interethnic variability (Wang et al., 2010; Appell et al., 2013). The population investigated in this study is characterized for being largely admixed with African Americans, Asians and Hispanics accounting for almost 30% of all individuals. As previously described, frequency of alleles ^*^3A, ^*^3B and ^*^3C is population-specific. Whereas ^*^3A and ^*^3B were predominantly found in Caucasians and Hispanics (3.36 and 2.90%, for ^*^3A and 0.07 and 0.1%, for ^*^3B, respectively), the most prevalent defective allele in African Americans and Asians was ^*^3C (4.57 and 2.21%, respectively). These results were similar to frequencies previously reported for those populations (Oliveira et al., 2007; Taja-Chayeb et al., 2008; Appell et al., 2013). Regarding TPMT associated-phenotypes, African Americans had the highest proportion of intermediate (10.31%) and low methylators (0.33%), being the ethnic group with the highest risk of developing adverse events derived from TPMT treatment. Conversely, Asians are the lowest risk group, with only 5.4% of individuals carrying one (5.30%) or two alleles (0.12%). Caucasians and Hispanics had a similar percentage of individuals with TPMT intermediate (7.54 and 8.15%, respectively) and low activity (0.30 and 0.33%, respectively). Other population-specific alleles not imputed in the current study, such as ^*^2 that is almost restricted to Caucasians, or ^*^6 (rs75543815) and ^*^8 (rs56161402) that occur at frequencies between 1.5 and 3.5% in some African and Asian populations (Oliveira et al., 2007), are also important contributors of TPMT deficiency. These rare *TPMT* alleles or novel variants will not be detected with this approach and can only be identified by direct genotyping or sequencing. Thus, the frequency of intermediate and low methylators in this study may be slightly underestimated.

It is worth mentioning that approximately 1 in 10 individuals tested from our biobank were found to carry at least one high-risk *TPMT* allele. There are currently over 2.5 million children enrolled in the CHOP healthcare system, and if one extrapolates the results yielded from this study to the entire population at CHOP, then more than 170,000 patients would be expected to be TPMT deficient. Identification of such carriers is especially important in the pediatric population, as thiopurines are commonly prescribed drugs in children. Thiopurines are the backbone drugs for maintenance of acute lymphoblastic leukemia (ALL), which is the most common childhood malignancy (Pui and Evans, 2006), and are also frequently used as chronic immunosuppressive therapy after organ transplantation and in inflammatory bowel disease (Dubinsky, 2004; Relling et al., 2011; Appell et al., 2013). A major limitation of their use is their narrow therapeutic index and the severe myelosuppression they cause, a life threating adverse event highly associated with *TPMT* deficiency (Relling et al., 1999). In a study by Relling and coworkers in 180 children with ALL receiving conventional doses of 6-mercaptopurin, the authors found that the cumulative incidence of toxicity was 100% for homozygous TPMT deficiency, 35% for heterozygous, and 7% for patients homozygous for allele ^*^1 (Relling et al., 1999). This association has been widely replicated, so genotyping of *TPMT* is recommended in US FDA-approved labeling and currently genotype-based dosing recommendations exist (Relling et al., 2011, 2013). The high genotype-phenotype correlation existent for TPMT and the large interethnic variability in the susceptibility to thiopurine hematopoietic toxicity, sustain the need of availing such genetic information to prospectively identify individuals where thiopurine therapy may need to be modified or changed.

Biorepositories where DNA samples are linked to the EMR of patients, such as the CHOP biobank, offer the ideal platform for screening and identification of individuals with high-risk genotypes that may require a modification in the therapy if a given drug is prescribed. CHOP is part of the Electronic Medical Records and Genomics (eMERGE) consortium that is actively working on large-scale testing and integration of information on actionable pharmacogenetic variants, such as *TPMT* alleles, into clinical practice using EMR technologies (Gottesman et al., 2013). One of the goals of eMERGE is the creation of SPHINX (Sequence, Phenotype, and pHarmacogenomics INtegration eXchange http://www.emergesphinx.org/), a web accessible repository of genomic variants derived from a panel of 84 genes involved in the pharmacogenetics of a large number of drugs, designed by the NIH-supported Pharmacogenetics Research Network (PGRN), and linked to clinical information. To date, SPHINX contains data on 2000 of the nearly 9000 subjects that are planned to be enrolled in the project. Interestingly, so far SPHINX lists 174 variants in the *TPMT* gene, including known and novel variants, and the minor allele frequency information. Variant repositories such as SPHINX allow the advance in the knowledge of pharmacogenetics through the exploration of new hypotheses and the further integration of this information into the EMR.

The results yielded from this study demonstrate that imputation of *TPMT* alleles from existing genomic data is feasible and may be used as a first step in the screening of high-risk individuals for thiopurine drugs toxicity. Sensitivity, specificity, and predictive values of the imputation were over 90% in all cases, except for the positive predictive value of the imputation of homozygous subjects. Given that around 90% of the population is expected to have two fully functional *TPMT* alleles, being able to accurately identify such individuals based on existing genomic data yields 10% of the population to be screened for high-risk genotypes with direct genotyping methods. The positive and negative predictive values of 100 and 97.40%, respectively, obtained for the discrimination of individuals with the ^*^1/^*^1 genotype supports the potential utility of imputation in narrowing the target population where *TPMT* genotypes need to be determined. Further integration of such pharmacogenetic information into the EMR, with clinical decision support, may be used to aid clinicians prescribe therapies with the maximum risk-benefit ratio based on each individual's information.

### Conflict of interest statement

The authors declare that the research was conducted in the absence of any commercial or financial relationships that could be construed as a potential conflict of interest.
